# Bovine Adenovirus-3 pVIII Suppresses Cap-Dependent mRNA Translation Possibly by Interfering with the Recruitment of DDX3 and Translation Initiation Factors to the mRNA Cap

**DOI:** 10.3389/fmicb.2016.02119

**Published:** 2016-12-27

**Authors:** Lisanework E. Ayalew, Amrutlal K. Patel, Amit Gaba, Azharul Islam, Suresh K. Tikoo

**Affiliations:** ^1^Vaccine and Infectious Disease Organization – International Vaccine Centre, University of Saskatchewan, Saskatoon, SKCanada; ^2^Department of Veterinary Microbiology, University of Saskatchewan, Saskatoon, SKCanada; ^3^Vaccinology & Immunotherapeutics Program, School of Public Health, University of Saskatchewan, Saskatoon, SKCanada

**Keywords:** BAdV-3, pVIII, DDX3, cap-dependent mRNA translation

## Abstract

Earlier, targeting of DDX3 by few viral proteins has defined its role in mRNA transport and induction of interferon production. This study was conducted to investigate the function of bovine adenovirus (BAdV)-3 pVIII during virus infection. Here, we provided evidence regarding involvement of DDX3 in cap dependent cellular mRNA translation and demonstrated that targeting of DDX3 by adenovirus protein VIII interfered with cap-dependent mRNA translation function of DDX3 in virus infected cells. Adenovirus late protein pVIII interacted with DDX3 in transfected and BAdV-3 infected cells. pVIII inhibited capped mRNA translation *in vitro* and *in vivo* by limiting the amount of DDX3 and eIF3. Diminished amount of DDX3 and eIFs including eIF3, eIF4E, eIF4G, and PABP were present in cap binding complex in BAdV-3 infected or pVIII transfected cells with no trace of pVIII in cap binding complex. The total amount of eIFs appeared similar in uninfected or infected cells as BAdV-3 did not appear to degrade eIFs. The co-immunoprecipitation experiments indicated the absence of direct interaction between pVIII and eIF3, eIF4E, or PABP. These data indicate that interaction of pVIII with DDX3 interferes with the binding of eIF3, eIF4E and PABP to the 5′ Cap. We conclude that DDX3 promotes cap-dependent cellular mRNA translation and BAdV-3 pVIII inhibits translation of capped cellular mRNA possibly by interfering with the recruitment of eIFs to the capped cellular mRNA.

## Introduction

DDX3, a member of DEAD (Asp-Glu-Ala-Asp) box family of RNA helicases (Tarn and Chang, 2009), is a 73 kDa nucleo-cytoplasmic shuttling protein and is biologically active both in the nucleus and the cytoplasm (Lai et al., 2008; Lee et al., 2008). DDX3 is essentially involved in transcription, pre-mRNA splicing, mRNA export, translation, mRNA turn over (Rocak and Linder, 2004), and regulation of innate immunity (Oshiumi et al., 2010; Schröder, 2011). Even though there are conflicting reports about the role of DDX3 in cap-dependent translation, evidences over the years support the positive role of DDX3 in promoting translation initiation (Lai et al., 2008, 2010; Lee et al., 2008; Geissler et al., 2012; Shih et al., 2012). More recently, DDX3 is suggested to be involved in the activation of translation initiation of a subset of mRNAs that carry long (Lai et al., 2008) or an RNA stem loop at their 5′ UTR (Lai et al., 2008; Soto-Rifo et al., 2012). DDX3 may accomplish modulation of cellular mRNA translation by interacting with specific eukaryotic translation initiation factors like eIF4E and PABP (Shih et al., 2012), eIF2α (Lai et al., 2008), eIF3 (Lee et al., 2008), or eIF4G (Soto-Rifo et al., 2012). Similarly, there is a strong evidence for the active involvement of Ded1, the yeast homologue of DDX3 in translation initiation (Chuang et al., 1997) and interacting with eIF4G (Hilliker et al., 2011). More recently, ATP dependent activation of translation initiation and interactions with the components of the translation machinery is established for Ded1. However, the translation of stalled mRNAs *in vitro* is repressed by Ded1 (Hilliker et al., 2011).

Adenovirus pVIII is a core protein, which stabilizes the virion structure by connecting the core of the virus with the inner surface of adenovirus capsid (Rohn et al., 1997). The pVIII is expressed as 24 kDa protein in bovine adenovirus 3 (BAdV-3) infected cells, which localizes to the cytoplasm and the nucleus of virus infected cells using classical importin α/β dependent nuclear import pathway (Ayalew et al., 2014). The pVIII is cleaved at amino acid 111 and 146 by BAdV-3 encoded protease (Ayalew et al., 2014). The cleaved C-terminus (amino acid 147–216) of BAdV-3 pVIII appears to interact with hexon and is incorporated in mature infectious virions (Ayalew et al., 2014).

Analysis of interaction of viral proteins with cellular proteins has not only helped in defining the role of cellular proteins in virus replication cycle but also their role in cellular processes. For instance, interaction of vaccinia virus K7 protein with DDX3 revealed the role of DDX3 in TBK1/IKKε mediated IRF activation (Schroder et al., 2008). The interaction of viral proteins with cellular proteins is of vital importance in the regulation of virus replication, growth and survival. One of these processes involves the translation of mRNAs. Different viruses are evidenced in utilizing various strategies to inhibit translation of capped cellular mRNA to facilitate their life cycle. For example, Rubella virus capsid protein inhibits protein translation by sequestration of PABP (Ilkow et al., 2008), picornaviruses like FMD virus induce inhibition of cap-dependent protein synthesis by cleaving, eIF4A and eIF4G (Belsham et al., 2000). Correspondingly, caliciviruses inhibit host cell cap dependent translation by cleaving eIF4GI and eIF4GII (Willcocks et al., 2004). Likewise, adenovirus infection facilitates its replication by altering cellular architecture and host cell gene expression (Hodge and Scharff, 1969; Puvion-Dutilleul et al., 1994) including inhibition of transport of cellular mRNAs (Beltz and Flint, 1979) and inhibition of translation of cellular capped mRNAs (Huang and Schneider, 1991). Although the cellular processes affected by adenoviruses are understood, little is known about the identity of the proteins (viral or cellular) and the mechanisms involved (Yueh and Schneider, 1996).

Lately, DDX3 is getting utmost medical importance on account of its significance in the development of cancer (Botlagunta et al., 2008; Bol et al., 2013) and the life cycle of important pathogens; including vaccinia virus (Schroder, 2010), hepatitis B virus (Wang and Ryu, 2010; Yu et al., 2010), hepatitis C virus (Breiman et al., 2005; Ariumi et al., 2007; Oshiumi et al., 2010), and human immunodeficiency virus (HIV)-1 (Yedavalli et al., 2004; Liu et al., 2011). Moreover, analyzing the targeting of DDX3 by a viral protein has defined the new role of DDX3 in the induction of innate immunity (Schroder et al., 2008).

However, the exact role of DDX3 in cap dependent translation is not clear. While some reports indicate that DDX3 acts as a general translation initiation factor (Lee et al., 2008; Geissler et al., 2012), another report indicates that DDX3 promotes the translation of a subset of selected mRNAs with structured mRNAs at their 5′ end (Soto-Rifo et al., 2012). In addition, Ded1 (the yeast homologue of DDX3) have been shown to promote general translation initiation by enhancing the formation and resolution of an eIF4F-mRNA complex (Hilliker et al., 2011).

Here, we provide evidence for the role of DDX3 in cap dependent mRNA translation and report that the interaction of DDX3 with BAdV-3 pVIII alters the translation of cellular mRNAs by interfering with recruitment of eukaryotic initiation factors to the cap binding complex without affecting the stability of mRNAs.

## Materials and Methods

### Cell Lines and Viruses

African green monkey kidney (Vero) cells (ATCC CCL-81) were grown in Dulbecco’s Modified Eagle’s (DMEM; Sigma Aldrich) medium containing 10% heat inactivated fetal bovine serum (FBS) (Sera Care Life Sciences, Inc.). 293T cells (ATCC CRL-11268) and Madin-Darby Bovine Kidney (MDBK) cells (ATCC CCL22) were grown in minimum essential medium (MEM; Sigma Aldrich) containing 5% FBS. Wild type BAdV-3 was used to infect MDBK cells at an MOI of 5.

### Antibodies

Anti-pVIIIa recognizes a protein of 24 kDa in BAdV-3 infected cells (Ayalew et al., 2014). Anti-DBP recognizes a protein of 48 kDa in BAdV-3 infected cells (Zhou et al., 2001). Anti-DDX3, anti-eIF4G, anti-eIF4E, anti-eIF3, anti-PABP and fluorescence conjugated goat anti-mouse IgG-FITC (Santa Cruz Biotechnology, Inc., USA), Cy-3 conjugated goat anti-rabbit antibody (Jackson Immuno Research), Alexa Flour 680 goat anti-rabbit IgG antibody (Molecular Probes) or IRDye 800 conjugated goat anti-mouse IgG (Li-COR biosciences) and anti-HA and anti-β-actin MAb (Sigma Aldrich) were purchased.

### Plasmid Construction

Plasmids were constructed (Supplementary File) using standard procedures.

### Recombinant Protein Expression and Protein Purification

The recombinant GST-fusion proteins were expressed in *Escherichia coli* BL21 cells as described (Kulshreshtha and Tikoo, 2008). Glutathione S-transferase (GST), GST.pVIII, and GST.DDX3 fusion proteins were purified using Glutathione sepharose beads (GE Healthcare) as per the instructions of the manufacturers. The purified proteins were dialyzed using Slide-A-Lyzer dialysis cassette (Thermo Scientific). The concentrations of the proteins were measured by Bradford assay (Bio Rad) using Ultrospec^®^ 3000 spectrophotometer (Pharmacia Biotech).

### *In vitro* Translation and Co-immunoprecipitation Assay

Individual plasmid DNAs (0.8 μg) were used to synthesize [^35^S] (Perkin Elmer) labeled human adenovirus (HAdV)-5 pVIII (pC.pVIIIhav) and porcine adenovirus (PAdV)-3 pVIII (pC.pVIIIpav) proteins or unlabeled DDX3 (pcHA.DX3) *in vitro* by utilizing a TNT T7 Coupled Reticulocyte Lysate System (Promega). Equal amounts of proteins (unlabeled DDX3 and labeled HAdV-5 pVIII or unlabeled DDX3 and labeled PAdV-3 pVIII) were mixed together and incubated for 4–6 h at 4°C. The individual mixture was immuno-precipitated either with anti-DDX3 serum or rabbit pre-immune sera coupled to protein A sepharose beads. The bound proteins were separated by 10% sodium dodecyl sulphate (SDS)-polyacrylamide gel electrophoresis (PAGE), fixed in de-stain solution for 30 min and dried. Subsequently, the gel was exposed to a phosphor screen (Kodak) and visualized on a Molecular Imager FX using Quantity One software (Bio-Rad).

### *In vitro* Binding Assay

Plasmid pHA.DX3 DNA (0.8 μg) was used to synthesize radio-labeled DDX3 protein *in vitro* by utilizing a TNT T7 Coupled Reticulocyte Lysate System (Promega) in the presence of 30 μCi of [^35^S]-methionine (Perkin Elmer). Prior to use, glutathione sepharose (GST) beads were washed three times with GST binding buffer (0.54 M NaCl, 2.7 mM KCl, 10.15 mM Na2HPO4, 1.75 mM KH2PO4, 0.01 M MgCl2, 1 μg/ml each Aprotinin and Leupeptin, 1% Triton X-100, 1 mM PMSF and DNAse I). Purified GST, GST.pVIII, or GST.DDX3 fusion protein (15 μg each) was incubated individually with 20 μl of glutathione sepharose beads (GE Health Care) plus 10 μl of *in vitro* synthesized indicated proteins at +4°C on a nutator. After overnight incubation, the beads were washed three times, 10 min each with 0.1 M PBS. The bound proteins were analyzed as described above.

### Immunoprecipitation and Western Blot

Madin-Darby Bovine Kidney cells were infected with BAdV-3 at an MOI of 5. Vero cells (6 × 10^5^/well) in one well of six well plate were co-transfected with 2 μg of each plasmid (pHA.DX3 and pEY.pVIII or pHA.DX3 and pEYFPN1) DNA. At 36 h post infection or 48 h post transfection, the cells were lysed, immunoprecipitated with indicated protein specific antibodies, separated by 10% SDS-PAGE, transferred to PVDF membrane and probed by Western blot using indicated protein specific antibodies as described (Poulin et al., 1998). Similarly, DDX3 knockdown 293T cells were transfected with 2 μg of pHA.DX3 or pHA.pVIII or pHA.DX3 and pc.pVIII. 48-h post transfection, the cells were lysed and precipitated using anti-HA affinity matrix (Sigma) and Western blot was performed as indicated above.

### Confocal Microscopy

Monolayers (60–70% confluent) of Vero cells (5 × 10^4^/well) in two well chamber slides were incubated with Opti-MEM I reduced serum media (Gibco). After 2 h of incubation, the cells were transfected with indicated plasmid DNA(s) (1–2 μg/plasmid DNA/well) using Lipofectamine 2000 (Invitrogen). After 36 h post transfection, the cells were fixed with 3.7% paraformaldehyde, permeabilized with 0.05% triton X-100 and blocked with 2% goat serum. Subsequently, the cells were incubated with protein specific antibody in 0.1 M PBS for 1 h at room temperature, followed by specific secondary antibody for 1 h. Finally, the slides were mounted with mounting medium containing DAPI (Vectashield) and visualized under Zeiss LSM 5 laser scanning con-focal microscope.

Monolayers (90% confluent) of MDBK cells (1 × 10^5^cells/well) grown on two well chamber slides were mock infected or infected with wild type BAdV-3 at an MOI of 5. At 24 h post infection, the cells were fixed with 3.7% paraformaldehyde, immunostained and visualized as described above.

### *In vitro* Synthesis and Translation of Capped and Uncapped mRNA

Firefly luciferase (FLuc) mRNAs were synthesized *in vitro* in the absence (uncapped) or presence (capped) of 40 mM Ribo m7GpppG cap analog (Promega) using T7-RiboMax RNA production system. Ten microliters of prewashed GST-beads were loaded with 750 ng of either GST.pVIII or GST proteins and incubated for 2 h at +4°C with 15 μl of rabbit reticulocyte lysate. Subsequently, the mixtures were centrifuged and the supernatants were used for the translation of the synthesized capped and uncapped mRNAs. Finally, luciferase assay (Promega) was performed in a luminometer as per the company’s procedure.

### Dual Luciferase Assay

To examine the effect of pVIII on the translation of IRES or Cap dependent translation, a bicistronic luciferase assay was performed in 293T cells using a plasmid DNA expressing a single transcriptional unit in which translation of renilla luciferase is cap-dependent while translation of FLuc is cap-independent (IRES dependent). The cells grown in six well plates were co transfected with 2 μg/well of a bicistronic reporter plasmid pcDNA3-RLuc-POLIRES-FLuc and 4 μg/well of either plasmid pEY.pVIII DNA or plasmid pEYFP N1 DNA. At 48 h post transfection, FLuc and Renilla reniformis luciferase (RLuc) activities were measured in a luminometer by using a dual luciferase assay kit (Promega) as per the company’s procedure. Expression of EYFP was used to normalize the transfection efficiency.

### Cellular Protein Synthesis Assay

The monolayers of MDBK cells in six well plates were mock infected or infected with BAdV-3 at an MOI of 5 or monolayers of Vero cells grown in six well plates were transfected with indicated concentrations of plasmid pEY.pVIII or pEYFPN1. At different times post transfection, the cells were starved in media without methionine for 2 h before labeling with 100 μCi of [^35^S] methionine for 10 min. The radiolabelled cells were collected and lysed with RIPA buffer containing protease inhibitor cocktail. Proteins from radiolabelled cell lysates were separated by 10% SDS-PAGE. The gel was fixed, exposed to a phosphor screen (Kodak) overnight and visualized on a Molecular Imager FX using Quantity One software (Bio-Rad).

### Analysis of DDX3 and eIFs *In vitro*

To determine whether the reduction in the level of the translation of capped luciferase mRNA in the presence of pVIII is due to the reduction in the level of eIFs, initially 10 μl of Flexi rabbit reticulocyte lysate was incubated with 10 μl of GST bead containing 750 ng of either GST or GST.pVIII fusion protein at +4°C and centrifuged for 10 min. Finally, the supernatant and pellet were collected and analyzed by Western blot using anti-DDX3 or anti-eIF specific antibodies, followed by Alexa Flour 680 goat anti-rabbit IgG (Molecular Probes) or IRDye 800 conjugated goat anti-mouse IgG (Li-COR biosciences) as secondary antibody. The membranes were scanned using Odyssey LiCOR infrared scanning system and the intensity of the bands measured by Odyssey Software v2.1.

Similarly, 50 μl of the cytoplasmic fraction of cells were incubated with 10 μl of GST beads (loaded with 750 ng of either GST or GST.pVIII fusion protein) at +4°C and centrifuged for 10 min. Finally, the samples were analyzed by Western blot as described above.

### 7-Methyl Guanosine Cap Binding Assay

The indicated cells grown on six well plates were infected with five MOI of BAdV-3. 293T cells grown on six well plates were transfected either with 2 μg/well of plasmid pEYFPN1 or pEY.pVIII DNAs. At 36 h post infection or transfection, the cells were lysed with RIPA buffer containing protease inhibitor cocktail and centrifuged at 10,000 rpm. The supernatants were incubated with 50 μl of 7-methyl GTP sepharose beads on a nutator overnight at +4°C. The beads were then washed three times 5 min each and boiled with protein loading dye with 10% beta mercaptoethanol. Proteins were separated by 10%SDS-PAGE gel, transferred to nitrocellulose and probed in Western blot using anti-DDX3 or anti-eIF specific antibody followed by Alexa Flour 680 goat anti-rabbit IgG (Molecular Probes) or IRDye 800 conjugated goat anti-mouse IgG (Li-COR biosciences) as secondary antibody. The expression of pVIII in BAdV-3 infected cells or EY.pVIII in transfected cells was checked by Western blot using anti-pVIII serum.

### Electrophoretic Mobility Shift Assay (EMSA)

Confluent MDBK cells grown in six well plates were starved with media without phosphate for 1 h before radiolabelling with 30 μci of [^32^P] UTP. After labeling for 1 h, the cytoplasmic RNA was extracted using SurePrep^TM^ nuclear or cytoplasmic RNA purification kit (Fisher Bioreagents). Finally, poly A^+^ RNA was purified using Oligotex^®^ mRNA mini kit (Qiagen). The purity of the RNA was further checked by digestion with either RNase or DNase enzymes followed by gel electrophoresis. Subsequently, RNA EMSA was carried out using a modified protocol by Yakhnin et al. (2012). Radiolabelled (10,000 cpm) RNA probe of oligo dT purified polyA^+^ RNA was incubated with 500 ng of GST alone, or GST fusion proteins GST.pVIII or GST.100K (BAdV-3 100K; used as positive control) at room temperature. After incubation for 30 min, the reaction mixture was separated by 4% acrylamide native gel for 4–6 h at 150 V before the gel was dried, exposed onto a phosphor screen (Kodak) and scanned on a Molecular Imager FX using Quantity One software (Bio-Rad).

## Results

### Interaction of BAdV-3 pVIII with Cellular Proteins

**FIGURE 1:**
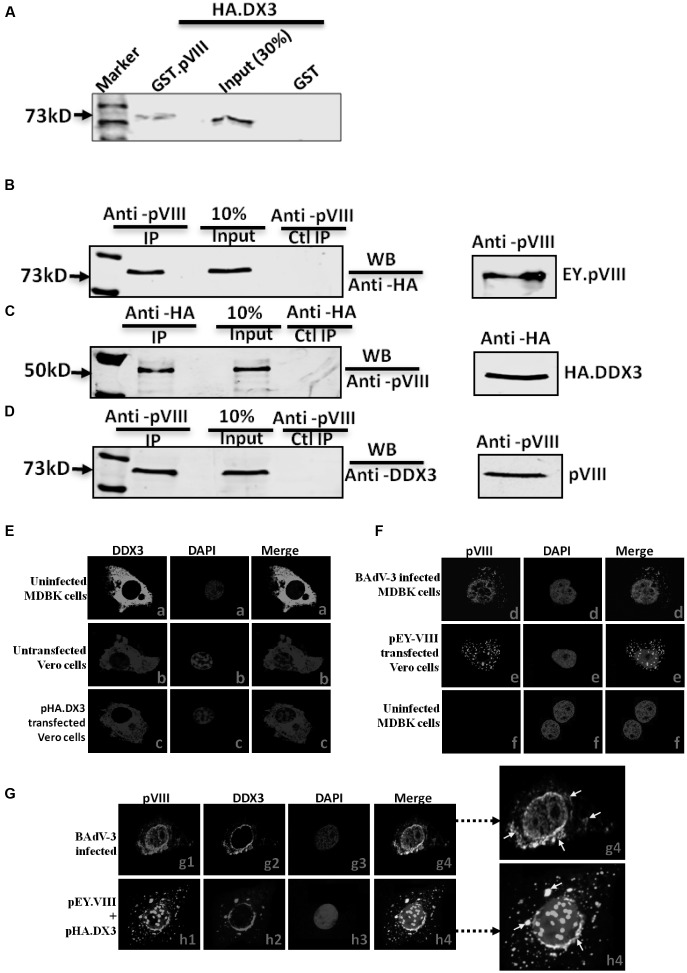
**Interaction of DDX3 with BAdV-3 pVIII.** **(A)** Glutathione S-transferase (GST) pull down assay. Purified GST or GST.pVIII fusion protein immobilized on Glutathione-Sepharose 4B beads, incubated with *in vitro* translated [^35^S] methionine labeled HA tagged DDX3 were separated by 10% SDS-PAGE and detected by autoradiography. **(B,C)** Co-immunoprecipitation in transfected cells. Proteins from the lysates of cells co-transfected with either pHA.DX3 and pEY.pVIII or pHA.DX3 and pEYFPN1 were immunoprecipitated with anti-pVIII serum **(B)** or anti-HA MAb **(C)**, separated by 10% SDS-PAGE and transferred to nitrocellulose membrane. The separated proteins were probed in Western blot using anti-HA MAb **(B)** or anti-pVIII serum **(C)**. **(D)** Co-immunoprecipitation in BAdV-3 infected cells. Proteins from the lysates of mock or BAdV-3 infected Madin-Darby Bovine Kidney (MDBK) cells were immunoprecipitated with anti-pVIII serum, separated by 10% SDS-PAGE, transferred to nitrocellulose membrane and probed in Western blot using anti-DDX3 MAb. Immunoprecipitation (IP). WB (Western blot). Ctl (Control)**. (E–G)** Confocal microscopy. MDBK cells mock infected (panels a and f) or infected with BAdV-3 (panels d and g1–g4) VERO cells untransfected (panel b) or transfected with indicated plasmid (panels c, e, and h1–h4) DNA, were fixed 36 h post-infection/transfection. The subcellular localization of DDX3 (panels a–c, g2, and h2) protein was visualized by indirect immunofluorescence (panels a–c, g2, h2) using anti-DDX3 MAb and fluorescein conjugated goat anti-mouse IgG-FITC (panels a and g2), anti-DDX3 MAb and Cy3 conjugated goat anti-mouse (pane b) secondary antibody, anti-HA MAb and Cy3 conjugated goat anti-mouse secondary antibody (panel c and h2). The subcellular localization of pVIII (panels d, e, f, g1, and h1) was visualized by direct fluorescence (panels e and h1) or indirect immunofluorescence using anti-pVIII serum and Cy3 conjugated goat anti-rabbit secondary antibody (panels d, f, and g1). Nuclei were stained with DAPI in each panel. A merge of the images is shown. Enlargement of panel g4 and h4 is shown, arrows in white shows few of the colocalization of pVIII and DDX3.

Initially, proteomic analysis of the nucleoli of BAdV-3 infected and uninfected MDBK cells identified a number of nucleolar proteins, which appeared to be involved in BAdV-3 infection (Patel and Tikoo, unpublished data). Since one of these proteins identified as DDX3 appeared to be important for BAdV-3 replication, we initially determined if DDX3 interacted with any BAdV-3 protein using matchmaker GAL4 Yeast two hybrid assays (Supplementary file). Our results suggested that BAdV-3 pVIII interacted with DDX3 (Supplementary Figure 1). The interactions were confirmed by GST-pull down assay (**Figure 1A**) and co immunoprecipitation assay using transfected cells (**Figures 1B,C**). Moreover, using co-immunoprecipitation coupled with Western blot analysis, we determined the interaction of DDX3 with pVIII in BAdV-3 infected MDBK cells. As seen in **Figure 1**, a 73 kDa DDX3 protein could be co-immunoprecipitated from the lysates of BAdV-3 infected MDBK cells using anti-pVIII serum and probed in Western blot with anti-DDX3 MAb (panel D).

Finally, confocal microscopy analysis revealed that pVIII co-localized with DDX3 around the perinuclear region and in the cytoplasm of the transfected cells (**Figure 1G**, panels h1–h4) or BAdV-3 infected cells (**Figure 1G**, panels g1–g4). The perinuclear localization may probably correspond to rough endoplasmic reticulum, the site of mRNA translation. However, DDX3 localized diffusely in the cytoplasm in uninfected MDBK or untransfected Vero cells (**Figure 1E**, panels a and b). The DDX3 also localized diffusely in the cytoplasm of transfected cells (**Figure 1E**, panel c). Additionally, pVIII localized both in the cytoplasm and in the nucleus in BAdV-3 infected (**Figure 1F**, panel d) or pEY.VIII transfected (**Figure 1F**, panel e) cells. No pVIII was detected in uninfected MDBK cells (**Figure 1E**, panel f).

### Interaction of DDX3 with pVIII of HAdV-5 and PAdV-3

**FIGURE 2:**
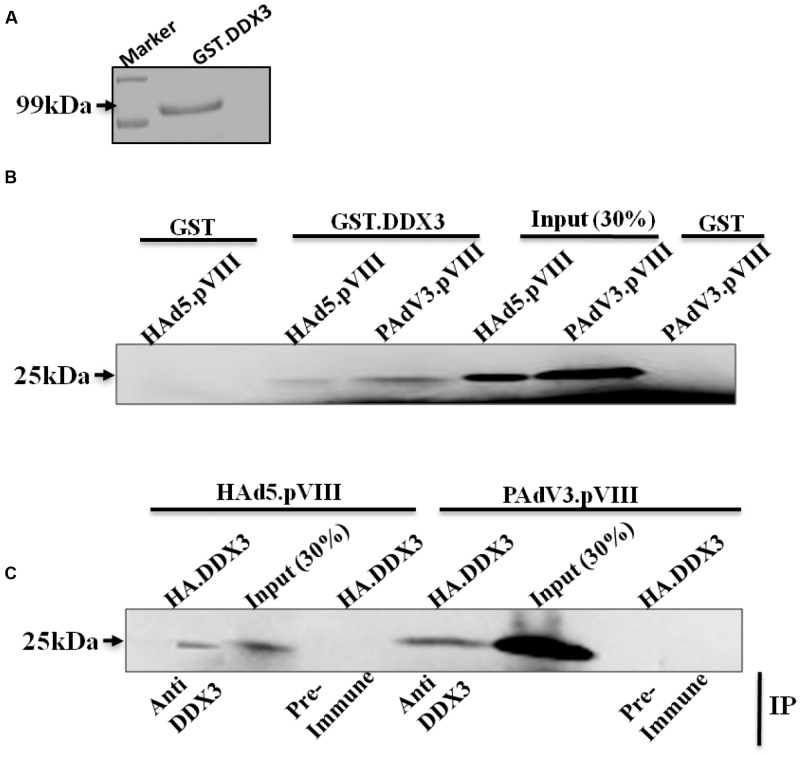
**Interaction of DDX3 with PAdV-3 and HAdV-5 pVIII.** **(A)** Coomassie blue staining of purified protein. Purified GST.DDX3 protein was separated by 10% SDS-PAGE and stained with 0.25 Coomassie blue stain. **(B)** GST-pull down assay. Purified GSTor GST.DDX3 fusion protein immobilized on Glutathione-Sepharose 4B beads, incubated individually with *in vitro* translated [^35^S] methionine labeled PAdV-3 pVIII or HAdV-5 pVIII, separated by 10% SDS-PAGE and detected by autoradiography. **(C)** Co-immunoprecipitation. Radio labeled *in vitro* transcribed and translated HAdV5 pVIII or PAdV-3 pVIII was incubated with *in vitro* transcribed and translated unlabeled DDX3 protein. Proteins were immunoprecipitated with either anti-DDX3 serum or rabbit pre immune sera, separated by 10% SDS-PAGE and auto radio-graphed. Immunoprecipitation (IP).

To verify if interaction of DDX3 with pVIII is conserved among members of *Mastadenovirus* genus, GST-pull down assay was performed using purified GST or GST.DDX3 fusion protein (**Figure 2A**). As seen in **Figure 2B**, radiolabelled HAdV-5 pVIII protein interacted with GST-DDX3 fusion protein but not with GST. Similarly, radiolabelled PAdV-3 pVIII protein (lanes c, e, and f) interacted with GST-DDX3 fusion protein but not with GST protein alone.

Next, we performed co-immunoprecipitation assay to corroborate the results of GST pull down assay. As seen in **Figure 2C**, radiolabelled HAdV-5 pVIII co-immunoprecipitated with unlabeled DDX3 using anti-DDX3 serum. Similarly, radiolabelled PAdV-3 pVIII co-immunoprecipitated with unlabeled DDX3 using anti-DDX3 serum. No such proteins could be co-immunoprecipitated using pre immune sera.

### BAdV-3 pVIII Affects Cellular Protein Synthesis

**FIGURE 3:**
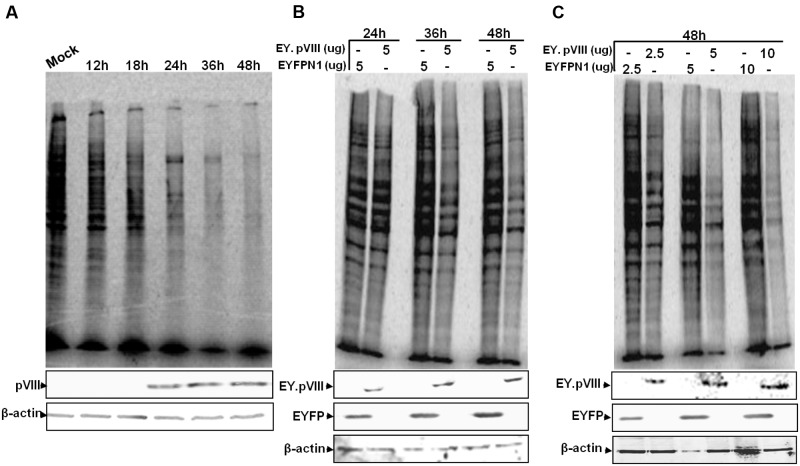
**Protein synthesis in BAdV-3 infected cells.** **(A)** Monolayers of MDBK cells were mock infected or infected with BAdV-3 at a MOI of 5. **(B,C)** Monolayers of VERO cells were transfected with indicated amounts of plasmid DNAs. At indicated times post infection **(A)** or transfection **(B,C),** the cells were pulse labeled with [^35^S] methionine for 10 min. The radiolabelled proteins were separated by 10% SDS-PAGE and analyzed by autoradiography. Proteins from the lysates of radiolabeled cells **(A,B or C)** were subjected to SDS-PAGE and Western blot using anti-pVIII serum, or anti-β-actin MAb.

To determine if BAdV-3 infection inhibits the synthesis of cellular proteins at late times post infection, mock or BAdV-3 infected MDBK cells were pulsed for short duration with [^35^S] methionine at different times post infection. Proteins from the lysates of the labeled cells were separated by 10% SDS-PAGE and analyzed by autoradiography. As seen in **Figure 3A**, compared to mock infected MDBK cells, reduction in cellular protein synthesis is apparent in BAdV-3 infected cells at 18 h post infection, which coincides with the detection of pVIII (a late viral protein) in BAdV-3 infected cells. By 36–48 h post infection, there is significant decrease in cellular protein synthesis, which coincides with steady expression of pVIII protein (**Figure 3A**).

To determine if expression of pVIII alters the cellular protein synthesis, Vero cells were transfected with indicated plasmid DNA, labeled with [^35^S] methionine, and analyzed by SDS-PAGE. As seen in **Figure 3B**, there was noticeable reduction in the synthesis of cellular protein in cells transfected with plasmid pEY.pVIII (express EYFP-pVIII fusion protein; Supplementary file) DNA than in cells transfected with plasmid pEYFP-N1 (express EYFP; Supplementary file) DNA at 24–48 h post transfection. Moreover, the reduction in cellular protein synthesis appeared to depend on the amount of plasmid DNA used for transfection (**Figure 3C**).

### BAdV-3 pVIII Affects Translation of Capped mRNAs

**FIGURE 4:**
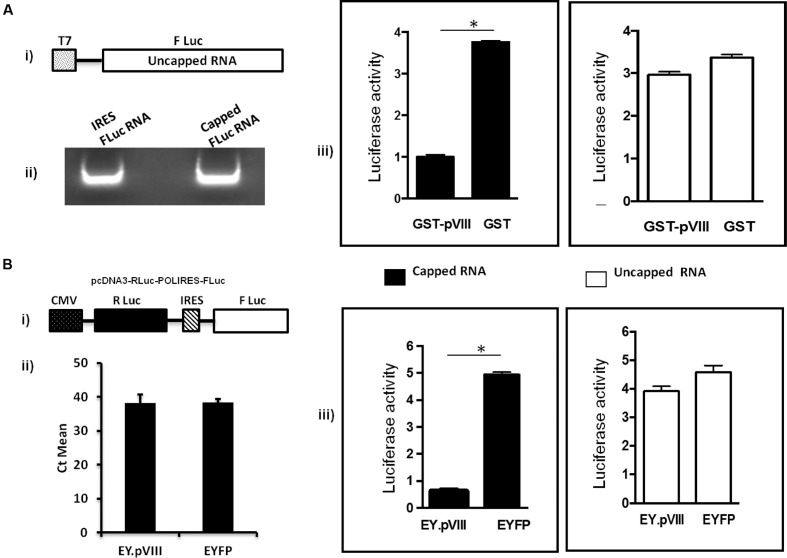
**Effect of pVIII on capped mRNA translation.** **(A).** *In vitro*. The TNT^®^ T7 luciferase DNA (Promega) (i) was transcribed *in vitro* in the absence (uncapped) or presence (capped) of 40 mM Ribo m7GpppG cap analog (Promega) using RiboMAX RNA production system-T7 (Promega). The *in vitro* synthesized capped and uncapped luciferase mRNAs (ii) were translated in the supernatant collected after centrifugation of mixture of Flexi Rabbit Reticulo Lysate (Promega) incubated with Glutathione sepharose beads preloaded with GST.VIII or GST protein alone. The level of luciferase activity was measured using a luciferase kit (Promega) on a Luminometer (Turner Designs, Inc.). The results are shown as relative luciferase activity (iii). Error bars indicate SE of means for separate experiments. The relative luciferase intensity is determined based on GST compared to GST.pVIII. **(B)** *In vivo*. 293T cells were transfected with plasmid DNAs (2 μg of pcDNA3-RLuc-POLIRES-FLuc (i) and either 4 μg of pEY.pVIII or 4 μg of pEYFPN1). At 36 h post transfection, Firefly luciferase (FLuc) and Renilla reniformis luciferase (RLuc) activities were measured in a luminometer by using a dual luciferase assay kit (Promega) as per the company’s procedure. Expression of EYFP was used to normalize the transfection efficiency. The results are shown as relative luciferase activity (iii). The level of cytoplasmic RLuc-POLIRES-FLuc mRNA both in EY.pVIII and EYFP expressing plasmid transfected cells was quantified by RT-PCR (ii). Error bars indicate SE of means for three separate experiments. ^∗^statistically significant.

To determine the effect of pVIII on mRNA translation, capped or uncapped FLuc mRNAs were synthesized (**Figure 4A**) and translated in the presence of purified GST or fusion protein; GST.pVIII. (Supplementary file). As seen in **Figure 4B**, GST.pVIII protein significantly inhibited the *in vitro* translation of capped, but not of uncapped luciferase mRNA. No such effect could be observed in the presence of GST on the translation of capped or uncapped luciferase mRNAs. No significant difference could be observed using different concentration of GST.pVIII fusion protein (Supplementary Figure 2).

To examine the effect of BAdV-3 pVIII on the translation of capped and uncapped mRNA translation *in vivo*, a dual luciferase assay was performed. As shown in **Figure 4B**, in the presence of EY.pVIII (Supplementary file), the cap dependent translation of renilla luciferase mRNA (capped) was significantly reduced compared to IRES dependent translation of FLuc mRNA (uncapped) in 293T cells. In contrast, there was no significant difference in the translation of renilla luciferase (cap-dependent) mRNA or FLuc (IRES dependent) in 293T cells in the presence of EYFP protein (**Figure 4B**). Moreover, cytoplasmic RLuc-POLIRES-FLuc mRNA levels appeared similar in EY.pVIII or EYFP expressing plasmid transfected 293T cells (**Figure 4B**).

### pVIII Protein Interacts with eIFs Indirectly via Its Interaction with DDX3

**FIGURE 5:**
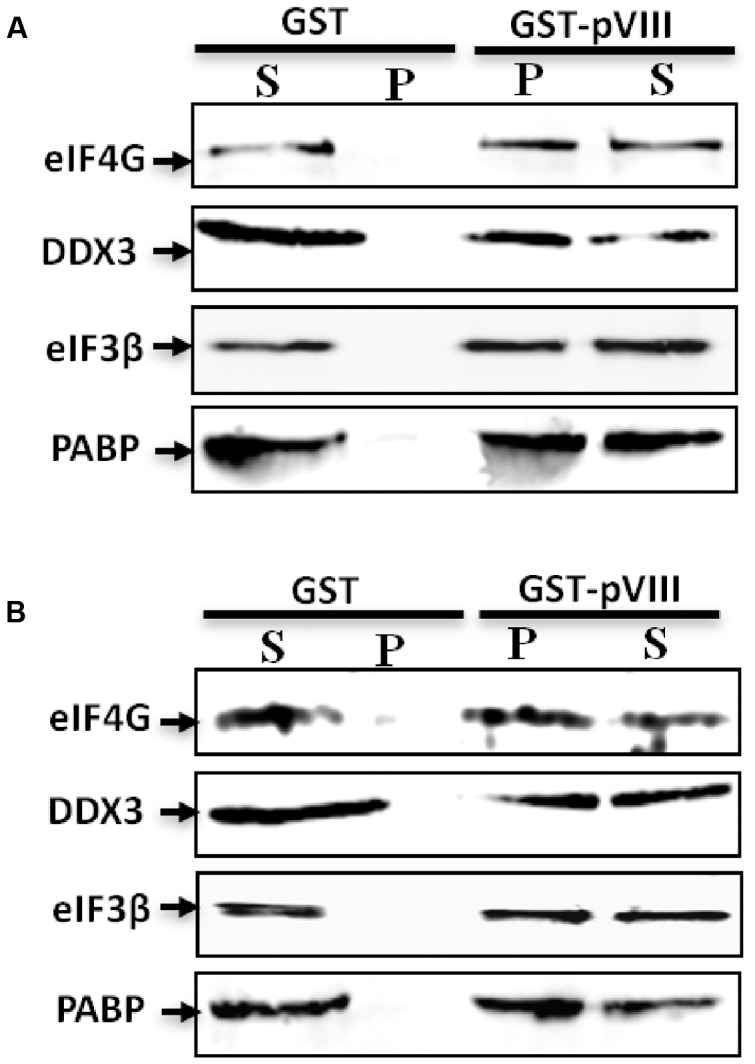
**The effect of pVIII on levels of eIFs.** The cytoplasmic fraction of MDBK cells (50 μl) or Flexi Rabbit Reticulo-Lysate (10 μl) were incubated with either 10 μl of GST beads loaded with 750 ng of purified GST.VIII protein or 10 μl of GST beads loaded with 750 ng of purified GST protein for 2 h at +4°C and centrifuged for 10 min. The supernatant (S) and the pellet (P) from both reticulo lysate **(A)** or cytoplasmic fraction **(B)** were separated by 10% SDS-PAGE gel and analyzed by Western blot using indicated protein specific antibodies and Alexa Flour 680 goat anti-rabbit IgG antibody or IRDye 800 conjugated goat anti-mouse IgG as secondary antibody.

To determine if the interaction of pVIII and DDX3 also affects the level of eIFs, the pellet and supernatant fractions of the rabbit-reticulo Lysates incubated with glutathione sepharose beads preloaded with purified GST.pVIII or GST were analyzed by Western blot using protein specific antibodies. As seen in **Figure 5A**, addition of GST.pVIII in reticulo lysate leads to the detection of DDX3, eIF3β, eIF4G, and PABP in the pellet fraction. No detection of DDX3, eIF3, eIF4G, or PABP could be observed in the pellet fraction when purified GST alone was added to the reticulo Lysate (**Figure 5A**). Similarly, addition of GST.pVIII in cytoplasmic fraction of MDBK cells leads to the detection of DDX3, eIF3β, eIF4G, and PABP in pellet fraction (**Figure 5B**). No detection of DDX3, eIF3β, eIF4G, and PABP could be observed in the pellet fraction when purified GST alone was added to cytoplasmic fraction of MDBK cells (**Figure 5B**). Thus, we conclude that pVIII forms immunoprecipitable complex with eIFs.

### BAdV-3 Infection or pVIII Expression Interferes with the Binding of DDX3 and Translation eIFs to 5′-Cap of mRNAs

**FIGURE 6:**
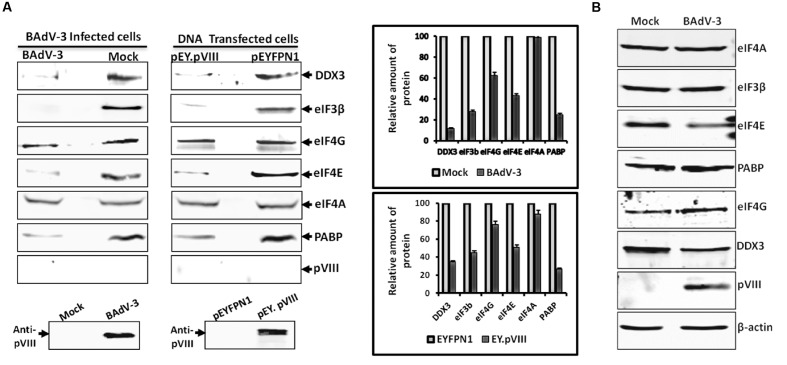
**m7GTP-sepharose binding assay.** **(A)** The supernatant of the lysates of the cells collected at 36 h post BAdV-3 infection of MDBK cells (mock or BAdV-3) or transfection of 293T cells with plasmid DNAs (pEY.pVIII or pEYFPN1) were incubated with m7GTP sepharose cap affinity beads. After washing, the bound proteins were analyzed by Western blot using indicated protein specific antibodies and IRDye 800 conjugated goat anti-mouse IgG or Alexa Flour 680 goat anti-rabbit IgG as secondary antibody. The intensity of the bands of the Western blot in all cases was analyzed by Odyssey Software v2.1. The relative amount of proteins in BAdV-3 infected or pEY.VIII transfected cell lysates that are retained in the 7-methyl GTP resins as compared to mock infected or pEYFPN1 transfected cells, respectively (i.e., considering the amount of protein in mock infected or pEYFPN1 transfected cell lysates that are retained in the m7GTP resins as 100%) is plotted. Error bars indicate SE of means for three separate experiments. Proteins from the lysates of BAdV-3 infected or transfected cells were separated by 10% SDS-PAGE and probed in Western blot using anti-pVIII serum. **(B)** Proteins from the lysates of mock infected or BAdV-3 infected MDBK cells collected at 36 h post infection were separated by 10% SDS-PAGE and analyzed by Western blot using protein specific antibody and anti-rabbit IRDye 800 conjugated goat anti-mouse IgG (Li-COR biosciences) or Alexa Flour 680 goat anti-rabbit IgG as secondary antibody. β-actin was used as a loading control.

To determine the effect of BAdV-3 on the association of eIFs with 5′-Cap of mRNAs, a Cap (m5GpppG) binding assay was performed using cleared lysates of BAdV-3 infected cells. The captured components of cap-binding complex were identified by Western blot using protein specific antibodies. As seen in **Figure 6A**, no significant difference could be observed in the binding of eIF4G and eIF4A to 5′-Cap (m7GpppG) in mock or BAdV-3 infected cells. However, significant decrease in the binding of DDX3, eIF3, eIF4E, and PABP proteins to 5′-Cap (m7GpppG) was observed in BAdV-3 infected cells compared to mock infected cells. However, pVIII was not retained in the m7GTP resins. To confirm for virus infection of cells, Western blot was performed on BAdV-3 infected cell lysates using anti-pVIII serum which detected the expression of pVIII.

Similarly, no significant difference could be observed in the binding of eIF4A to 5′-Cap (m7GpppG) in the cells (**Figure 6A**) transfected with plasmid pEY.pVIII or plasmid pEYFP.NI (Supplementary file) DNA. However, there was significant decrease in the binding of DDX3, eIF3, eIF4E, eIF4G, and PABP proteins to 5′-Cap (m7GpppG) in the cells transfected with plasmid pEY.pVIII DNA than the cells transfected with plasmid pEYFP.N1 (Supplementary file) DNA. Interestingly, pVIII was not retained in the 7-methyl GTP resins. Moreover, anti-pVIII serum detected the expression of EY.pVIII fusion protein in the transfected cells (**Figure 6A**). The quantified intensity of the protein bands were plotted to show the relative amount of each protein retained in the m7GTP-Sepharose beads as compared to the total amount. In addition, to determine if BAdV-3 infection degrades eIFs, VIDO R2 cells were mock infected or infected with BAdV-3 and analyzed by Western blot 36 h post infection. As seen in **Figure 6B**, significant level of PABP, eIF3β, eIF4G, DDX3, and eIF4E could be detected in BAdV-3 infected cells. β-actin was used as a loading control.

### pVIII Does Not Interact Directly with eIFs

**FIGURE 7:**
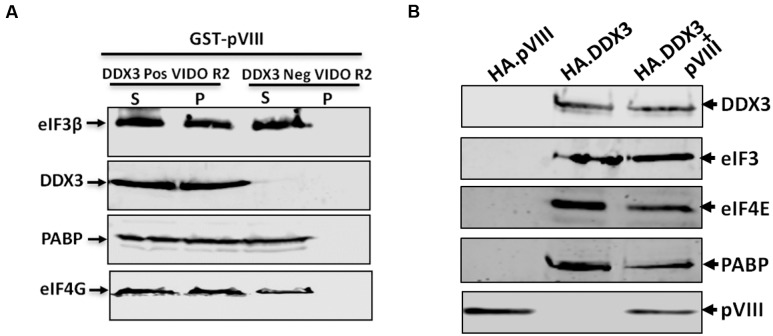
**Interaction of pVIII with eIFs.** **(A)** The cytoplasmic fraction (50 μl) of DDX3 positive or negative VIDO R2 cells were incubated with 10 μl of beads loaded with 750 ng of purified GST-VIII protein for 2 h at 4°C and centrifuged for 10 min. The supernatant (S) and the pellet (P) from both cytoplasmic fraction were separated by 10% SDS-PAGE gel and analyzed by Western blot using indicated protein specific antibodies and IRDye 800 conjugated goat anti-mouse IgG or Alexa Flour 680 goat anti-rabbit IgG as secondary antibody. Total amount of indicated protein in cytoplasmic fraction of indicated cells was estimated by Western blot analysis before adding GST.pVIII fusion protein. **(B)** DDX3 knockdown 293T cells were transfected with 2 μg of pHA.DX3 or pHA.pVIII or pHA.DX3 and pc.pVIII. The cells were lysed at 48 h post transfection and precipitated by anti-HA affinity matrix and the precipitates were analyzed by Western blot as indicated above.

To determine if pVIII interacts directly with eIFs, the cytoplasmic fractions of VIDO R2 cells (Reddy et al., 1999) and DDX3 knocked down VIDO R2 cells (Ayalew and Tikoo, in preparation) were collected, incubated with purified GST or GST.VIII, centrifuged to collect supernatant and pellet fractions and analyzed by Western blot. As seen in **Figure 7A**, addition of GST.pVIII lead to the detection of DDX3, eIF3β, eIF4G, and PABP in the pellet of DDX3 positive VIDO R2 cell cytoplasmic fractions. No such detection of DDX3, eIF3β, eIF4G, and PABP could be observed in the pellet fraction when purified GST.VIII was added to the cytoplasmic fraction of DDX3 knocked down VIDO R2 cells. Similar results were observed when HA.pVIII, HA.DDX3 or HA.DDX3 plus pVIII expressing plasmid transfected DDX3 knocked down 293T cells were immunoprecipitated using anti-HA affinity matrix. The expression of HA.pVIII did not co-immunoprecipitate the eIFs in DDX3 knockdown 293T cells. In contrast, expression of HA.DDX3 or HA.DDX3 and pVIII co-immunoprecipitated eIFs in DDX3 knockdown 293T cells (**Figure 7B**).

### Cellular Protein Synthesis in DDX3 Silenced Bovine Cells

**FIGURE 8:**
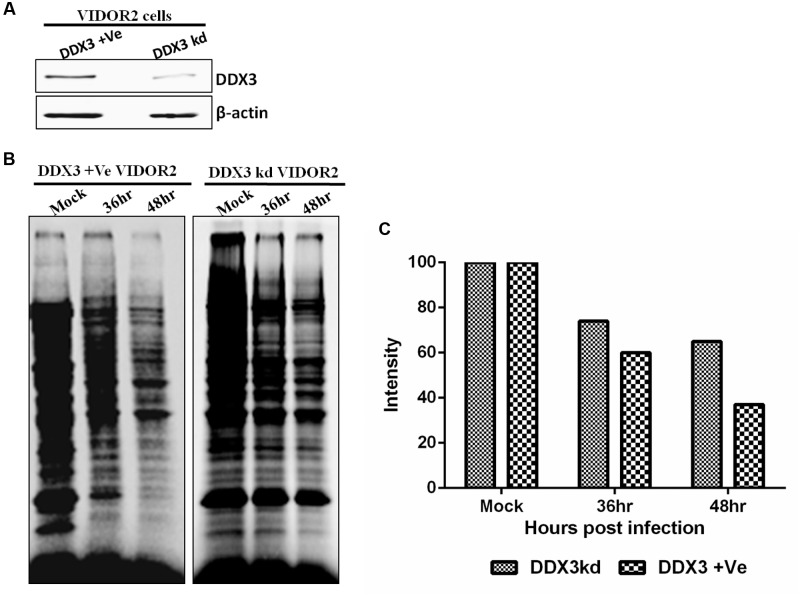
**Protein synthesis in BAdV-3 infected DDX3kd VIDOR2 cells.** **(A)** Western blot of lysates of DDX3 +Ve or DDX3kd VIDOR2 cells with anti-DDX3 antibody. **(B,C)** [^35^S] methionine pulse labeling and quantification. Mock infected or BAdV-3 infected cells were pulse labeled with [^35^S] methionine for 10 min at 36 h or 48 h post infection. The radiolabelled proteins were separated by 10% SDS-PAGE and analyzed by autoradiography. **(C)** The intensity of each lane was quantified using GelQuant.NET software and plotted relative to the intensity of the mock infected cell lanes.

To determine if the silencing of DDX3 in BAdV-3 permissive VIDO R2 cells affects the cellular protein synthesis, [^35^S] methionine pulse labeling experiment was performed using mock or BAdV-3 infected DDX3 +Ve VIDO R2 (DDX3 positive) or DDX3kd VIDO R2 (DDX3 knockdown) (**Figure 8A**) cells. As seen in **Figures 8B,C**, there was significant difference in the synthesis of cellular proteins between mock infected and BAdV-3 infected VIDO R2s cells at 36–48 h post infection. In contrast, there was less inhibition of synthesis of cellular proteins in DDX3 kd VIDO R2 cells as compared to DDX3 +Ve VIDOR2 cells. This further strengthens the critical importance of DDX3 in suppression of cellular protein synthesis in BAdV-3 infected cells.

### pVIII Does Not Directly Bind to Poly A RNA

To assess the possibility of the interaction of pVIII with RNA, ^32^P (UTP) labeled Poly A^+^ containing mRNA was purified from the cytoplasmic fraction of MDBK cells and RNA electrophoretic mobility shift assay was performed using purified GST alone or fusion protein GST.pVIII. Since adenovirus 100K protein interacts with mRNAs (Cuesta et al., 2004), RNA electrophoretic mobility shift assay was also performed using GST.100K as a positive control. As seen in Supplementary Figure 3, GST.pVIII did not interact with the cellular poly A RNA. Similarly, no interaction was observed between the cellular polyA^+^ RNA and GST. As expected (Cuesta et al., 2004), GST.100K interacts with cellular polyA mRNA.

### Cytoplasmic Cellular mRNA Levels Are Not Affected in BAdV-3 Infected Cells

**FIGURE 9:**
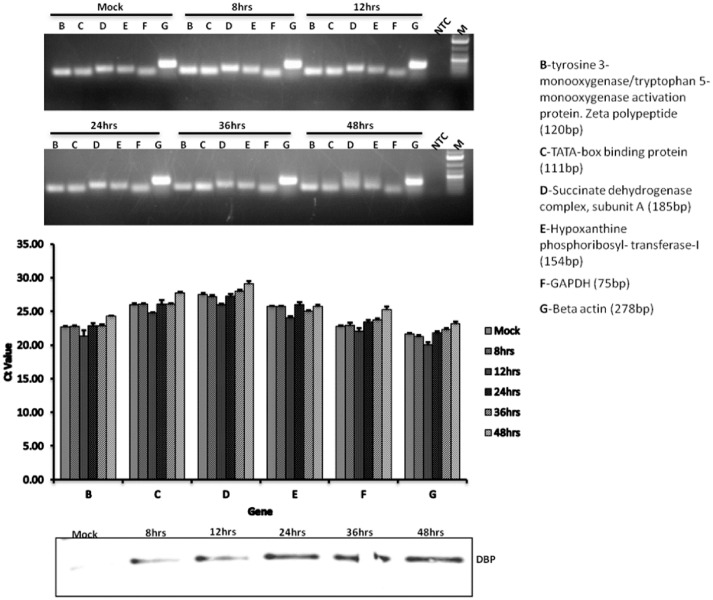
**qRT-PCR.** MDBK cells were infected with BAdV-3 at an MOI of 5. At indicated times post infection cytoplasmic RNA was purified and cDNA synthesized and qRT-PCR performed using specific primers targeting the indicated bovine housekeeping genes as described in the materials and methods. Western blot was performed using anti-BAdV-3 DBP (DNA binding protein) to confirm productive infection. Error bars indicate S.E of means for three separate experiments.

To examine whether cytoplasmic mRNA levels are affected or their stability compromised, cDNAs were synthesized from cytoplasmic mRNAs purified from mock infected cells or BAdV-3 infected cells collected at different time points post infection. Real time PCR (Supplementary file) was performed using primers targeting different species of bovine housekeeping genes (Lisowski et al., 2008). The results indicate that (**Figure 9**) as compared to mock infected cells, the level of cytoplasmic mRNAs is not altered in BAdV-3 infected cells even at 48 h post infection Moreover, the integrity of cellular mRNAs appears intact during BAdV-3 virus infection. The detection of expression of DNA binding protein in BAdV-3 infected cell lysates by Western blot using anti-DBP serum (Zhou et al., 2001) confirm virus infection.

## Discussion

Like human adenovirus-5 (Huang and Schneider, 1991; Yueh and Schneider, 1996), global cellular protein synthesis was inhibited at late times post BAdV-3 infection, which appears to be linked with the activity of adenovirus late protein(s). Since DDX3 acts to promote cap dependent translation initiation (Geissler et al., 2012; Soto-Rifo et al., 2012), the interaction of DDX3 with pVIII may interfere with translation of capped dependent mRNAs. Several observations support this speculation. First, the inhibition of cellular protein synthesis observed at late times post infection correlates with the expression of pVIII in BAdV-3 infected cells. Secondly, expression of pVIII in transfected cells alters the cellular protein synthesis in dose and time dependent manner. Thirdly, *in vitro* translation of only capped luciferase mRNAs is inhibited in the presence of purified GST-VIII, but not in the presence of GST alone. Fourthly, pVIII does not alter the mRNA stability or transport to the cytoplasm. Finally, translation of only capped renilla luciferase mRNA is inhibited in transfected cells expressing EY.pVIII but not in transfected cells expressing EYFP.

Translation of eukaryotic mRNAs involves the binding of mRNA cap (5′m^7^GpppN) structure to cap binding protein complex (eIF-4F), which include eIF4E, eIF4A, and eIF4G (Jackson et al., 2010) proteins. However, alterations in one or more eIFs may impair the cap dependent translation of cellular mRNAs (Jivotovskaya et al., 2006; Hinnebusch and Lorsch, 2012). A number of viruses inhibit host protein synthesis (Bushell and Sarnow, 2002; Schneider and Mohr, 2003) by modifying the activity of eukaryotic initiation factors, which are required for bringing ribosomes to capped mRNAs (Feigenblum and Schneider, 1993; Schroder, 2010), by sequestration of PABP (Ilkow et al., 2008), cleaving eIF4G and/or dephosphorylation of 4E-BP1 (Liu et al., 2011), or decreasing activity of eIF4E (Connor and Lyles, 2002; Burgui et al., 2007) by direct interaction of viral protein(s) with eIFs.

Earlier report suggested that direct binding of adenovirus 100K protein to eIF4G induces under phosphorylation of eIF4E by displacing Mnk1 leading to the inhibition of cap-dependent translation (Cuesta et al., 2000, 2004). A different strategy appears to be adopted by BAdV-3 pVIII protein to alter the host protein synthesis. Although eIFs are not degraded in BAdV-3 infected cells, the binding of eIFs to mRNA cap (5′m7GpppN) structure appears to be interfered in BAdV-3 infected or pVIII transfected cells. Absence of binding of pVIII to eIFs in DDX3 depleted cells suggest that pVIII does not appear to interact directly with eIFs. Since DDX3 interacts with translation initiation factors including eIF3, eIF4E, eIF4G, and PABP (Lai et al., 2008; Lee et al., 2008; Shih et al., 2008, 2012), the interaction of pVIII with DDX3 may affect the interaction of DDX3 along with associated eIFs with cap binding protein complex and thus inhibiting cellular protein synthesis in BAdV-3 infected cells. The synthesis of some cellular protein in BAdV-3 infected DDX3kd compared to BAdV-3 infected DDX3 + cells suggest that multiple mechanisms exist for the inhibition of cellular protein synthesis at late times post BAdV-3 infection.

Recent reports suggest that DDX3 promotes cap dependent translation initiation by directly interacting with eukaryotic initiation factor eIF3 in an RNA independent manner (Yedavalli et al., 2004; Lai et al., 2008; Lee et al., 2008; Geissler et al., 2012). The eIF3 is a multi-subunit mammalian initiation factor, which binds to 40S ribosomal subunit preventing premature joining of 40S and 60S ribosome subunits, and also interacts with eIF4G to help to recruit m^7^G capped mRNAs promoting the formation of 43S pre-initiation complex (Jackson et al., 2010). It is possible that interaction of pVIII with DDX3 sequesters both DDX3 and eIF3 modulating the efficient formation of 43S preinitiation complexes thus affecting the translation of capped mRNAs. Our results demonstrate that addition of pVIII cause significant reduction in the amount of available eIF3 and DDX3, thus limiting the translation of capped mRNAs *in vitro* or *in vivo*. Similarly, m^7^GTP Cap analog captured significantly reduced level of DDX3 and eIF3 from extracts of BAdV-3 infected or pVIII expressing plasmid DNA transfected cells. However, such preinitiation complexes are loaded on adenovirus late mRNAs (Yueh and Schneider, 1996). It is possible that interaction of BAdV-3 pVIII with DDX3 does not affect the formation of 43S pre initiation complex, but impairs the ability of eIF3 to interact with eIF4G thus abolishing the association of 43S complex with capped mRNAs. Vpg protein of Norwalk virus interacts with eIF3 and inhibits translation of capped mRNAs possibly by interfering with interaction of eIF4G with eIF3 (Daughenbaugh et al., 2003). Moreover, no pVIII was detected in m7GTP resins which rules out the possibility of the competitive binding of pVIII with m7GTP cap.

Earlier reports suggested that binding of adenovirus late protein 100K to eIF4G may displace kinase Mnk1, thus affecting the phosphorylation of translation eukaryotic initiation factor eIF4E (Cuesta et al., 2000, 2004). Under-phosphorylation of eukaryotic initiation factor eIF4E late (∼40 h) in adenovirus infected cells is thought to destabilize the interaction of cap structure of cellular mRNAs with eIF4F in 43S initiation complex (Huang and Schneider, 1991; Yueh and Schneider, 1996). Although reduced phosphorylation of eIF4E is usually associated with inhibition of cellular capped mRNAs translation (Huang and Schneider, 1991), the specific mechanism is still not clear.

Binding of adenovirus 100K protein to eIF4G does not affect the binding of eukaryotic initiation factors eIF4E, eIF4A, and PABP to cap initiation complex eIF4F *in vivo* (Cuesta et al., 2000). In contrast, interaction of DDX3 with pVIII significantly diminished the binding of not only eIF3 but also eIF4E, PABP or eIF4G to m7GTP cap. While PABP mediates binding of eIF4E and eIF4A to capped and polyadenylated mRNAs by interacting with eIF4G (Brook et al., 2012), eIF4E is the least abundant protein of translation initiation complex, thus it controls the rate of formation of eIF4F complex (Feigenblum and Schneider, 1993). It is possible that BAdV-3 infection sequesters PABP and/or eIF4E and affects the assembly of eIF4F complex in infected cells. Indeed, earlier reports suggest that limited availability of eIF4E (Feigenblum and Schneider, 1993; Piron et al., 1998) or PABP (Piron et al., 1998) in cap initiation complex, eIF4F inhibit translation of capped mRNAs.

## Author Contributions

LA, ST, and AP conceived and designed the experiments. LA, AP, AG, and IA performed the experiments. LA, ST, and IA analyzed the data. LA, ST, and AG wrote the paper.

## Conflict of Interest Statement

The authors declare that the research was conducted in the absence of any commercial or financial relationships that could be construed as a potential conflict of interest.
