# Supplementary material for: Bovine Adenovirus-3 pVIII Suppresses Cap-Dependent mRNA Translation Possibly by Interfering with the Recruitment of DDX3 and Translation Initiation Factors to the mRNA Cap
Source: Front Microbiol. 2016 Dec 27;7:2119. doi: 10.3389/fmicb.2016.02119 (PMC5186766; doi:10.3389/fmicb.2016.02119)
Supplement: Supplementary file 1 [file Presentation_1.PDF]

Supplementary information

Interaction of DDX3 and Bovine adenovirus-3 pVIII modulate the cellular mRNA translation by excluding eIFs from cap binding complex

Materials and methods

Plasmid Construction

Plasmid pcDNA3-RLuc-POLIRES-FLuc (1) was a gift from Dr. Nahum Sonenberg, McGill University, Montreal, Canada. Plasmid pcDDX-3 was a gift from Dr. Arvind H. Patel, MRC-University of Glasgow, Center for Virus Research, University of Glasgow, UK. and plasmid pcDNA3.1(neo)3HA DNA was a gift from Dr. Joyce Wilson, University of Saskatchewan. The construction of plasmid pGEX-100K expressing GST.100K fusion protein has been described (2). Plasmid GC.linker and plasmid GN.linker were a gift from Dr. Abraham Loyter, Hebrew University, Jerusalem.

a) *pcDNA.3HA*. The cDNA coding for 3HA epitopes was amplified by PCR by using primers 3HA-F and 3HA-R (S Table 1), and plasmid pcDNA3.1(neo)3HA DNA as a template. The PCR product was digested with *Hind*III-*Eco*RI and ligated to *Hind*III-EcoRI digested plasmid pcDNA3.1 (-) (Invitrogen) creating plasmid pcDNA.3HA.
b) *pHA.DX3*. A 2035bp *Eco*RI-*Bam*HI fragment was isolated from plasmid pcDDX-3 and ligated to *Eco*RI -*Bam*HI digested plasmid pcDNA.3HA creating plasmid pHA.DX3.
c) *pEY.VIII.* A 660bp DNA fragment containing pVIII gene was amplified by PCR using primers VIII-F and VIII-R (S Table 1), and plasmid pFBAV302 (3) DNA as a template.

The PCR product was digested with *Eco*RI and ligated to *Afe*I (blunt end repaired with T4 polymerase) - *Eco*RI digested plasmid pEYFPN1 (Clontech) creating plasmid pEY.VIII.

d) *DDX3 yeast expression plasmids*. A 1938bp DNA fragment was amplified by RT-PCR using primers DDX3-F and DDX3-R (S Table1), and RNA (isolated from MDBK cells) as a template, digested with *Eco*RI-*Bam*HI and ligated to *Eco*RI-*Bam*HI digested plasmid pGBKT7 or pGADT7 creating plasmid pGB.DX3 and pGA.DX3, respectively.

e) *pVIII yeast expression plasmids*. A 660bp DNA fragment containing pVIII gene was amplified by PCR using primers VIII-F and VIII-R (S Table 1), and plasmid pFBAV302 (3) DNA as a template. The PCR product was digested with *Eco*RI and ligated to *Nde*I (blunt end repaired with T4 polymerase)-*Eco*RI digested plasmids pGBKT7 or pGADT7 creating plasmids pGB.pVIII and pGA.pVIII, respectively.

f) *pGST.pVIII.* A 666bp *Hind* III (blunt end repaired with T4 polymerase) - *Xho*I fragment of plasmid pR.pVIII was ligated to *Aat*II (blunt end repaired with T4 polymerase) - *Xho*I digested plasmid pGEX-5X-1 (GE Healthcare) creating plasmid pGST.pVIII.

i) *pGST.DDX3*. A 2062bp BamHI (blunt end repaired with T4 polymerase)-*Not*I fragment was isolated from pHA.DX3 and ligated to *Sal*I (blunt end repaired with T4 polymerase) -*Not*I digested plasmid pGEX-5X-2 (GE Health Care) creating plasmid pGST.DDX3.

j) *pC.pVIIIhav.* A 693bp fragment was amplified by PCR using primers hpVIII-F and hpVIII-R (S Table 1), and plasmid pH5-R (4) DNA as a template. The PCR product was digested with *Bam*HI - *Xho*I and ligated to *Bam*HI- *Xho*I digested plasmid pcDNA.3HA creating plasmid pC.pVIIIhav.

k) *pC.pVIIIpav*. A 681bp fragment was amplified by PCR using primers PpVIII-F and PpVIII-R (S Table 1), and plasmid pGADT7-pVIII (5) DNA as a template. The PCR fragment was digested with *Bam*HI- *Eco*RI and ligated to *Bam*HI-*Eco*RI digested plasmid pcDNA.3HA creating plasmid pC.pVIIIpav.

l) *pGN.pVIII.* A 648bp fragment was amplified by PCR using primers VIII XmaI-F and VIII SacII-R (Table 1), and plasmid pEY.pVIII DNA as a template. The PCR product was digested with XmaI - SacII and ligated to XmaI - SacII digested plasmid pGN-linker, in frame with the N-terminus of GFP gene creating plasmid pGN.pVIII.

m) *pGC.DDX3.* A 1896bp fragment was amplified by PCR using primers DDX3 XmaI F and DDX3 NotI R (Table 1), and plasmid pHA.DX3 DNA (unpublished) as a template. The PCR product was digested with XmaI - NotI and ligated to XmaI - NotI digested plasmid pGC-linker in frame with the C-terminus of GFP gene creating plasmid pGC.DDX3.

n) *pGC.DDX3d1*. A 396bp fragment was amplified by PCR using primers GCDDX3d1F and GCDDX3d1R (Table 1), and plasmid pGC.DDX3 DNA as a template. The PCR product was digested with XmaI - SacII and ligated to XmaI - SacII digested plasmid GC-linker in frame with the C-terminus of GFP gene creating plasmid pGC.DDX3d1.

o) *pGC.DDX3d2*. A 1200bp fragment was amplified by PCR using primers GCDDX3d2F and GCDDX3d2R (Table 1), and plasmid pGC.DDX3 DNA as a template. The PCR product was digested with XmaI - SacII and ligated to XmaI - SacII digested plasmid pGC-linker in frame with the C-terminus of GFP gene creating plasmid pGC.DDX3d2.

p) *pGC.DDX3d3.* A294bp fragment was amplified by PCR using primers GCDDX3d3F and GCDDX3d3R (Table 1), and plasmid pGC.DDX3 as a template. The PCR product was digested with XmaI - SacII and ligated to XmaI - SacII digested plasmid pGC-linker in frame with the C-terminus of GFP gene creating plasmid pGC.DDX3d3.

q) *pGN.pVIIId1.* A 216bp fragment was amplified by PCR using primers GNVIII d1F and GNVIIId1R (Table 1), and plasmid pGN.pVIII DNA as a template. The PCR product was digested with XmaI - SacII and ligated to XmaI - SacII digested plasmid pGN-linker, in frame with the N-terminus of GFP gene creating plasmid pGN.pVIIId1.

r) *pGN.pVIIId2*. A 231bp fragment was amplified by PCR using primers GNVIIId2 and GNVIIId2R (Table 1), and plasmid pGN.VIII DNA as a template. The PCR product was digested with XmaI - SacII and ligated to XmaI - SacII digested plasmid pGN-linker, in frame with the N-terminus of GFP gene creating plasmid pGN.pVIIId2.

s) *pGN.pVIIId3*. A 195bp fragment was amplified by PCR using primers GNVIII d3-F and GNVIIId3R (Table 1), and plasmid pGN.pVIII as a template. The PCR product was digested with XmaI - SacII and ligated to XmaI - SacII digested plasmid pGN-linker, in frame with the N-terminus of GFP gene creating plasmid pGN.pVIIId3.

Yeast II hybrid assay. The Matchmaker two hybrid system3 using Saccharomyces cerevisiae (Clonetech) was used to detect the interactions between pVIII and DDX3 as described (2). The pVIII gene was cloned in-frame to GAL4 DNA binding domain (pGB.pVIII) or GAL4 activation domain (pGA.pVIII). Similarly, DDX3 gene was cloned in-frame to GAL4 DNA binding domain (pGB.DX3) or in-frame to GAL4 activation domain pGA.DX3). Yeast AH109 cells (Clontech) were co-transformed with either pGA.DX3 or pGB.pVIII or pGA.pVIII and pGB.DX3 plasmid DNAs. The growth and selection of positive clones was performed as described earlier (2, 5). DNA was isolated from positive clones and sequenced for confirmation.

Real time PCR. MDBK cells were mock infected or infected with 5MOI of wild type BAdV-3. The cells were collected at different times post infection and cytoplasmic RNA was extracted using SurePrep^{TM} nuclear or cytoplasmic RNA purification kit. cDNA was synthesized using Superscript II (Invitrogen) followed by real time PCR using SYBR green and primers targeting different species of bovine housekeeping genes (6). pcDNA3-RLuc-POLIRES-FLuc and pEY.pVIII or EYFP NI plasmid transfected 293T

cells were collected at 36hrs post transfection and RNA was extracted using SurePrep^{TM} nuclear or cytoplasmic RNA purification kit. cDNA was synthesized using Superscript II (Invitrogen) followed by real time PCR using SYBR green and primers targeting RLuc-POLIRES-FLuc mRNA using primers Fir-F and Fir-R (S Table-1)

References

1. Poulin, F., Gingras, A., Olsen, H., Chevalier, S. and Sonenberg, N. (1998). 4E-BP3, a new member of the eukaryotic initiation factor 4E-binding protein family. *J. Biol. Chem.* 273, 14002-14007.
2. Kulshreshtha, V. and Tikoo, S.K. (2008). Interaction of bovine adenovirus-3 33K protein with other viral proteins. *Virology*. 381(1), 29-35.
3. Zakhartchouk, A.N., Reddy, P.S., Baxi, M., Bca-Estrada, M.E., Mehtali, M., Babiuk, L.A.Q. and Tikoo, S.K. (1998). Construction and characterization of E3-deleted bovine adenovirus type 3 expressing full-length and truncated form of bovine herpes type 1 glycoprotein gD. *Virology.* 250, 220-229.
4. Zakhartchouk, A.N., Pyne, C., Mutwiri, G., Papp, Z., Baca-Estrada, M.E., Griebel, P., Babiuk, L.A. and Tikoo, S.K. (1999). Mucosal immunization of calves with recombinant bovine adenovirus-3: Induction of protective immunity to bovine herpesvirus-1. *J. Gen. Virol.* 80, 1263-1269.
5. Singh, M., Shmulevitz, M. and Tikoo, S.K. (2005). A Newly identified interaction between IVa2 and pVIII proteins during porcine adenovirus type 2 infection. *Virology.* 336, 60-69.

6. Lisowski, P., Pierzchala, M., Goscik, J., Pareek, C.S., Zwierzchowski, L. (2008) Evaluation of reference genes for studies of gene expression in the bovine liver, kidney pitutary and thyroid. *J Appl Genet*. 49(4), 367-372.

Supplementary Table

S Table 1. List of primers

Primer name	Primer Sequence
**VIII-F**	5’-CATATGAGCAAAGAAATTCCCACAC
**VIII-R**	5’-GAATTCCGCTATAACCGCTCACAG
**DDX3-F**	5’-GCGAATTCATGAGTCATGTGGCGGTGG
**DDX3-R**	5’-GCGGATCCACGTTACCCCAGTCAAC
**3HA-F**	5’-ATAAAGCTTCGCCACCATGGATTACCCATACGATGTTCCTGAC
**3HA-R**	5’-CTAGAATTCGCCCGGGCCAGCGTAATCTGGAACGTCATATGG
**hp-VIII-F**	5’-GCGGATCCATGAGCAAGGAAATTCCCACGCCCTAC
**hpVIII-R**	5’-ATCTCGAGTCATCAGTCGTAGCCGTCCGCCGAGTC
**PpVIII-F**	5’-GCGGATCCATGAGCAAACAAATCCCCAC
**PpVIII-R**	5’-CGAATTCTCAGTCATAGCCTGCTACAGAGTCCG
**pVIII XmaI-F**	5’-AACAGGCCGCGGTCAGCTATAACCGCTCACAGAGTTG
**pVIII SacII-R**	5’-TATGACCCCGGGATGAGCAAAGAAATTCCCACACC
**DDX3 XmaI-F**	5’-AGTTTACCCGGGATGAGTCATGTGGCAGTGGAAAATGC
**DDX3 NotI-R**	5’-ATTATGCGGCCGCCAGTTACCCCACCAGTCAAC
**Fir-F**	5’- CGTGCCAGAGTCTTTCGACA
**Fir-R**	5’- ACAGGCGGTGCGATGAG
**VIII SacII-R**	5’ GCCGCGGTCAATCTTCATCTGATTTGTCACACCAGC

**DDX3 NotI R**	5’ GCCCGGGATGAGTCATGTGGCAGTGGAAA
**DDX3 XmaI F**	5’ GCCCGGGATGGATTGGTCAAAACCACTCCCAC
**VIII XmaI-F**	5’ GCCCGCGGTCATTTAGCTTCAACAAGAAGATCC
**GCDDX3d1F**	5’ AAAAAGGCCGCGGTCAGCTATAAC
**GCDDX3d1R**	5’ ATTCCGCGGTCAGTTACCCCACCAGTC
**GCDDX3d2F**	5’ GGATGTCGCCCCGGGATGAGCAAAGAAA
**GNVIIId3R**	5’ AACCGCGGTCATCTGGGATCAATCAG
**GCDDX3d3F**	5’ TTAACCCGGGATGCAGTGGCCGCCCACCTC
**GCDDX3d3R**	5’ TTCCGCGGTCAAGATGAACGCGAGCCTCCTCC
**GNVIIId1F**	5’ TACCCGGGATGTTCAACCCCCTGCAAA
**GNVIIId1R**	5’ GCCCGGGATGCAAGAAGTGCCGTCTTGGTTAG
**GNVIIId2F**	5’ AACAGGCCGCGGTCAGCTATAACCGCTCACAGAGTTG
**GNVIIId2R**	5’ TATGACCCCGGGATGAGCAAAGAAATTCCCACACC
**GNVIIId3F**	5’ AGTTTACCCGGGATGAGTCATGTGGCAGTGGAAAATGC
**GCDDX3d2R**	5’ ATTATGCGGCCGCCAGTTACCCCACCAGTCAAC
