# Supplementary material for: Bovine Adenovirus-3 pVIII Suppresses Cap-Dependent mRNA Translation Possibly by Interfering with the Recruitment of DDX3 and Translation Initiation Factors to the mRNA Cap
Source: Front Microbiol. 2016 Dec 27;7:2119. doi: 10.3389/fmicb.2016.02119 (PMC5186766; doi:10.3389/fmicb.2016.02119)
Supplement: Supplementary file 2 [file Presentation_2.PDF]

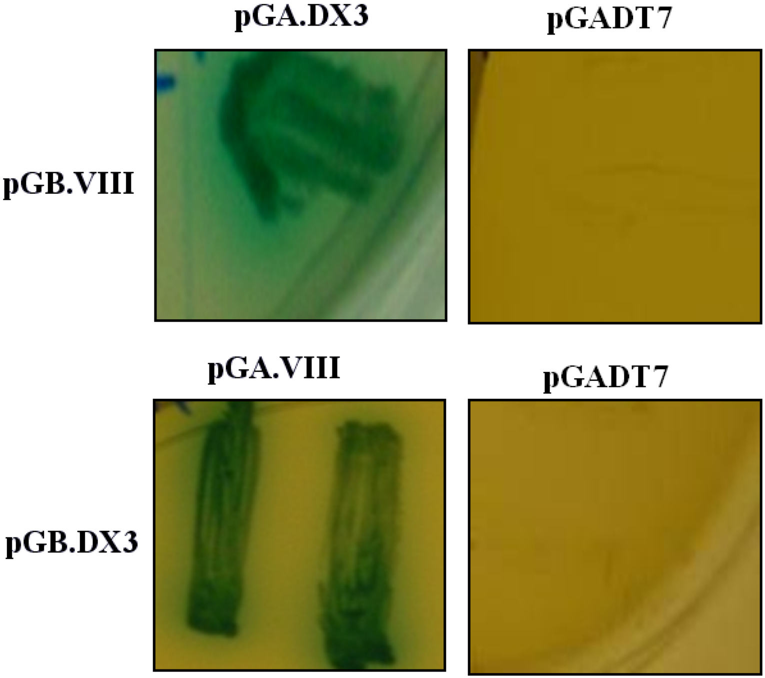

Fig. S1. Yeast two hybrid analysis. The AH109 yeast cells were co-transfected with plasmid (pGB.VIII and pGA.DX3; pGB.VIII and pGADT7; pGB.pDX3 and pGA.VIII or pGB.DX3 and pGADT7) DNAs and streaked on a selective medium containing X-α-gal but lacking Leu, Trp, His and Ade.
