# Supplementary material for: Bovine Adenovirus-3 pVIII Suppresses Cap-Dependent mRNA Translation Possibly by Interfering with the Recruitment of DDX3 and Translation Initiation Factors to the mRNA Cap
Source: Front Microbiol. 2016 Dec 27;7:2119. doi: 10.3389/fmicb.2016.02119 (PMC5186766; doi:10.3389/fmicb.2016.02119)
Supplement: Supplementary file 3 [file Presentation_3.PDF]

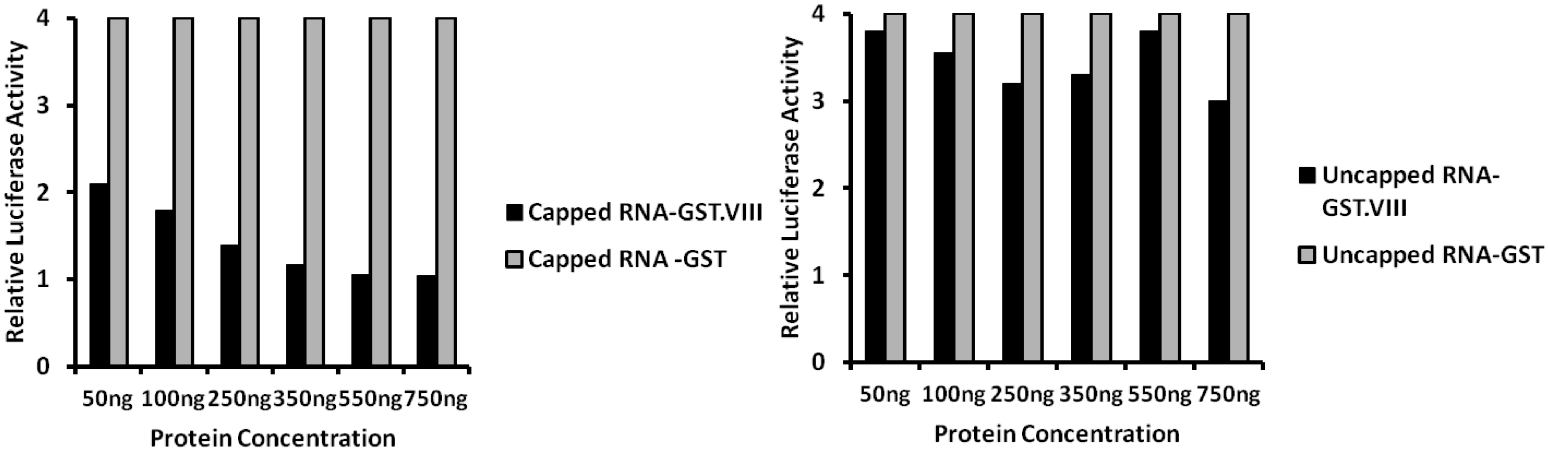

Fig. S2. Effect of different concentration of purified pVIII on capped mRNA translation *in-vitro*. The TNT® T7 luciferase DNA (Promega) was transcribed *in-vitro* in the absence (uncapped) or presence (capped) of 40mM Ribo m7GpppG cap analogue (Promega) using RiboMAX RNA production system-T7. The *in-vitro* synthesized capped and uncapped luciferase mRNAs were translated in the supernatant collected after centrifugation of mixture of Flexi Rabbit Reticulo Lysate incubated with Glutathione sepharose beads preloaded with GST.VIII or GST protein at different concentrations (50, 100, 250, 350, 550 or 750ngs). The level of luciferase activity was measured using a luciferase kit on a Luminometer. The relative luciferase activity of GST.VIII treated as compared to GST treated is plotted.
