# Supplementary material for: Bovine Adenovirus-3 pVIII Suppresses Cap-Dependent mRNA Translation Possibly by Interfering with the Recruitment of DDX3 and Translation Initiation Factors to the mRNA Cap
Source: Front Microbiol. 2016 Dec 27;7:2119. doi: 10.3389/fmicb.2016.02119 (PMC5186766; doi:10.3389/fmicb.2016.02119)
Supplement: Supplementary file 4 [file Presentation_4.PDF]

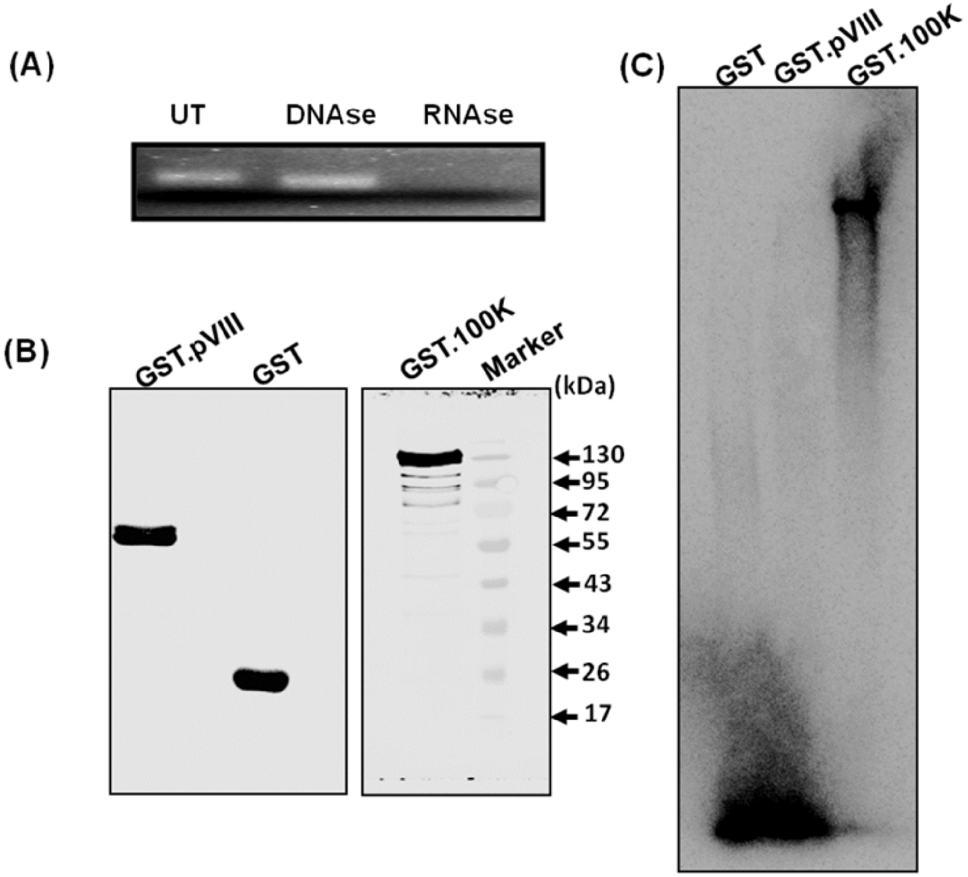

Fig. S3. Electrophoretic mobility Shift Assay. (A) Oligo dT column purified cellular polyA$^+$ RNA undigested (UN) or digested with either DNase or RNAse enzymes was analyzed by agarose gel electrophoresis. **(B)** Purified GST or GST fusion proteins were separated by 10% SDS-PAGE, transferred to nitrocellulose and probed in Western blot with anti-GST serum. The size of the molecular weight markers is shown on the right. **(C)** [^{32}P] (UTP) Labeled (10000cpm) poly A$^+$ containing cellular RNA purified from the cytoplasmic fractions of MDBK cells was incubated either with 500ng of GST alone, BAdV-3 pVIII fused to GST (GST.pVIII) or BAdV-3 100K fused to GST (GST.100K) fusion protein for 30min, separated by 4% acrylamide native gel and detected by autoradiography.
